# Liraglutide Ameliorates Erectile Dysfunction *via* Regulating Oxidative Stress, the RhoA/ROCK Pathway and Autophagy in Diabetes Mellitus

**DOI:** 10.3389/fphar.2020.01257

**Published:** 2020-08-12

**Authors:** Penghui Yuan, Delin Ma, Xintao Gao, Jiaxing Wang, Rui Li, Zhuo Liu, Tao Wang, Shaogang Wang, Jihong Liu, Xiaming Liu

**Affiliations:** ^1^Department of Urology, Tongji Hospital, Tongji Medical College, Huazhong University of Science and Technology, Wuhan, China; ^2^Hubei Institute of Urology, Tongji Hospital, Tongji Medical College, Huazhong University of Science and Technology, Wuhan, China; ^3^Department of Endocrinology, Tongji Hospital, Tongji Medical College, Huazhong University of Science and Technology, Wuhan, China

**Keywords:** liraglutide, diabetes mellitus, erectile dysfunction, oxidative stress, autophagy

## Abstract

**Background:**

Erectile dysfunction (ED) occurs more frequently and causes a worse response to the first-line therapies in diabetics compared with nondiabetic men. Corpus cavernosum vascular dysfunction plays a pivotal role in the occurrence of diabetes mellitus ED (DMED). The aim of this study was to investigate the protective effects of glucagon-like peptide-1 (GLP-1) analog liraglutide on ED and explore the underlying mechanisms *in vivo* and *in vitro*.

**Methods:**

Type 1 diabetes was induced in rats by streptozotocin, and the apomorphine test was for screening the DMED model in diabetic rats. Then they were randomly treated with subcutaneous injections of liraglutide (0.3 mg/kg/12 h) for 4 weeks. Erectile function was assessed by cavernous nerve electrostimulation. The corpus cavernosum was used for further study. *In vitro*, effects of liraglutide were evaluated by primary corpus cavernosum smooth muscle cells (CCSMCs) exposed to low or high glucose (HG)-containing medium with or without liraglutide and GLP-1 receptor (GLP-1R) inhibitor. Western blotting, fluorescent probe, immunohistochemistry, and relevant assay kits were performed to measure the levels of target proteins.

**Results:**

Administration of liraglutide did not significantly affect plasma glucose and body weights in diabetic rats, but improved erectile function, reduced levels of NADPH oxidases and ROS production, downregulated expression of Ras homolog gene family (RhoA) and Rho-associated protein kinase (ROCK) 2 in the DMED group dramatically. The liraglutide treatment promoted autophagy further and restored expression of GLP-1R in the DMED group. Besides, cultured CCSMCs with liraglutide exhibited a lower level of oxidative stress accompanied by inhibition of the RhoA/ROCK pathway and a higher level of autophagy compared with HG treatment. These beneficial effects of liraglutide effectively reversed by GLP-1R inhibitor.

**Conclusion:**

Liraglutide exerts protective effects on ED associated with the regulation of smooth muscle dysfunction, oxidative stress and autophagy, independently of a glucose- lowering effect. It provides new insight into the extrapancreatic actions of liraglutide and preclinical evidence for a potential treatment for DMED.

## Introduction

Erectile dysfunction (ED) is a common clinical entity in sexual dysfunction, influencing quality of life and self-confidence for men to a large extent. Diabetes mellitus (DM) is one of the most concerned diseases for developing ED. Diabetes mellitus ED (DMED) occurs at an early age, and is threefold more frequent in diabetic men compared with nondiabetic men, which affects 35–90% men with diabetes (Thorve et al., 2011). It is estimated that more than 300 million will experience diabetes globally by 2025 and 693 million by 2045 (Cho et al., 2018), along with increasing cases of DMED. Compared with nondiabetic men, patients with ED are inclined to be refractory to phosphodiesterase type 5 inhibitors, the first-line therapeutic drug for ED currently, due to incompletely understood mechanisms (Spitzer et al., 2012). Besides, although penile prosthesis implantation can be used as a supplement against the drug resistance, it is an invasive and expensive procedure, and DM increases the risk of infection and failure (Holland and Kohler, 2015). Therefore, the therapeutic manners of DMED remain insufficient, and it is necessary to further explore the pathophysiology of DMED and novel therapies.

Penile smooth muscle relaxation is the terminal process of erection with Ras homolog gene family (RhoA)/Rho-associated protein kinase (ROCK) signaling pathway involved (Shamloul and Ghanem, 2013). In diabetes, accumulation of advanced glycation end (AGEs) products contributes greatly to excessive production of reactive oxygen species (ROS) (Tang et al., 2019), subsequently inducing inflammation, interrupting cellular homeostasis, upregulating RhoA/ROCK pathway and damaging the corpus cavernosum smooth muscle function to a large extent (Kamenov, 2015; Wang et al., 2016), and all of these largely contribute to the pathogenesis of DMED. Therefore, it is significant to ameliorate the pathological process induced by oxidative stress for recovery of smooth muscle function.

Autophagy is a biological process against detrimental conditions such as oxidative stress and nutrient deprivation by recycling damaged organelles and cellular components to maintain cellular homeostasis (Filomeni et al., 2015). It is revealed that autophagy may play vital roles in diabetes-related diseases (Muratsubaki et al., 2017; Kong et al., 2018a), but the effects of autophagy in DMED are not illuminated enough.

Glucagon-like peptide-1 (GLP-1) is an incretin hormone released from intestinal L cells. It plays a crucial role in blood glucose regulation *via* the GLP-1 receptor (GLP-1R) (Jojima et al., 2017). GLP-1R agonists have been applied widely in the clinical treatment of diabetes nowadays. In addition to administration in glycemic control, accumulating evidence has implicated that GLP-1 exhibited various biological functions in extrapancreatic tissues. Studies showed that GLP-1R is expressed in the cardiovascular system, including endothelium and vascular smooth muscle cells (Tang et al., 2019). It has been reported that GLP-1 agonists show cardioprotective and vasodilatory effects (Hattori et al., 2010), such as ameliorating endothelial and vascular dysfunction, protecting against cardiac ischemia and steatosis, relieving atherosclerosis and left ventricular performance after myocardial infarction (Inoue et al., 2015). Besides, GLP-1 could exert its antioxidant effect to inhibit AGEs–induced increase of ROS in high glucose condition (Hendarto et al., 2012; Shi et al., 2015). As part of the systemic vasculature, it is significant to expound the function of GLP-1 in the corpus cavernosum. In this research, we show that GLP-1 analog liraglutide could improve erection function in DMED by regulating smooth muscle dysfunction, oxidative stress and autophagy, independently of a glucose-lowering effect in a diabetic rat model and corpus cavernosum smooth muscle cells (CCSMCs).

## Materials and Methods

### Ethical Approval

All protocols in this study were approved by the Experimental Animal Administration Committee of Wuhan Servicebio Biotechnology in China (NO.2019006).

### Experimental Animals

Forty male Sprague-Dawley (SD) rats (8 weeks old) purchased from Hunan Slack Jingda Experimental Animal Co., Ltd. were used in this study. After 7-day adaptive feeding with fasting overnight followed, the rat model of type 1 diabetes was constructed in 30 rats by injection of streptozotocin (STZ) (60 mg/kg; Sigma-Aldrich) - citric acid buffer as previously reported (Cui et al., 2018) and the rest 10 rats were treated with citric acid buffer and appointed in the control group. 3 and 7 days later, the level of blood glucose of all the rats was measured and rats with fasting blood glucose levels >16.7 mmol/L were regarded as diabetic state (Song et al., 2019).

Ten weeks later, 27 rats with diabetes survived. Then an apomorphine (APO) test was conducted to assess erectile function in rats. An APO (100 μg/kg) solution was injected subcutaneously into the necks of rats. Then the rats were placed in a dark and quiet room for 30 min, and the number of erections was observed and recorded. Once the glans protruded the foreskin, a score of 1 was recorded. A total score of 0 was regarded as DMED. Those with ED (n=20) according to the test were further divided into the DMED group (n=10) and the DMED + liraglutide (DMED + Lir) group (n=10) randomly. Rats in the DMED + Lir group were treated with liraglutide (Novo Nordisk) by subcutaneous injection at a dose of 0.3 mg/kg/12 h for 4 weeks due to short pharmacokinetic half-life (Sturis et al., 2003). And rats in the DMED group were treated with saline. It must be noted that animal group sizes had been defined before any data were obtained.

### Assessment of Erectile Function *In Vivo*

After the administration for 4 weeks, an electrostimulation test was conducted to evaluate the erectile function of all the rats (Yang et al., 2013). The rats were anesthetized by pentobarbital (40 mg/kg). Then the left carotid artery of rats was exposed and mean atrial blood pressure (MAP) was recorded continuously by intubation in the biological signal system. In the meanwhile, the corpus cavernosum was isolated and the cavernous nerves were exposed. By stimulating the nerves with a bipolar electrode (15 Hz frequency; 1.2 ms width; 5.0 V), the intracavernous pressure (ICP) was recorded. Then the erectile function was quantified by the maximal ICP (max ICP) and MAP (max ICP/MAP). After the measurement of erectile function, the rats were sacrificed and corpus cavernosum was dissected from the penile tissue. Then it was cut into three pieces after discarding the part inserted by the needle. The middle piece was maintained in paraffin for histologic studies and the remaining pieces were collected and stored at −80°C for ROS detection and other experiments.

### Cell Culture

The smooth muscle cells were prepared from the corpus cavernosum of normal male SD rats (8 weeks old) as previously described (Yang et al., 2018). After skin and mucosa were removed, the corpus cavernosum was cut and plated into a culture flask. These explants were cultured with low-glucose Dulbecco’s modified Eagle’s medium (DMEM) supplemented with 10% fetal bovine serum, 1% penicillin and streptomycin (Song et al., 2019). Then the cells were passaged and purified for subsequent experiments. CCSMCs were identified by α-smooth muscle actin (α-SMA) (1:200, GB13044, Servicebio, China) and desmin (1:200, GB12081, Servicebio) staining.

The cells from the fourth to the eighth passages were used in the experiments. CCSMCs were incubated with serum-free DMEM for 24h. Then cells were divided into four groups: control group (4.5 mM glucose); high-glucose (HG) group (33 mM glucose); HG + Lir group (33 mM glucose and 100 mM liraglutide) (Yu et al., 2018) and HG + Lir + Exe group [33 nM glucose, 100 mM liraglutide and 200 mM GLP-1 receptor antagonist, exendin (9-39)]. Liraglutide was added to the medium 60 min before HG treatment, and exendin (9–39) was added 30 min before HG treatment (Shi et al., 2015). Similar with studies *in vivo*, cell group sizes had been defined before any data were obtained.

### Western Blotting

The corpus cavernosal tissues and CCSMCs were processed in RIPA buffer supplemented with protease inhibitor cocktail and broken up using ultrasound furthermore. After that, the supernatant of protein was collected by centrifugation. The concentrations of total protein were obtained by a BCA assay kit (Boster Biological Technology).

Equal amounts of total protein from each sample were separated by electrophoresis on a 10%–12% SDS-PAGE gels and transferred to polyvinylidene difluoride (PVDF) membranes. After blocking with 5% fat-free milk in TBST, the target protein was incubated with primary antibodies against: nicotinamide adenine dinucleotide phosphate (NADPH) oxidase (NOX)1(1:500, ab131088, Abcam, USA), NOX2 (1:500, ab180642, Abcam), NOX4 (1:500, ab154244, Abcam), ROCK1 (1:1,000, 21850-1-AP, Proteintech, China), ROCK2 (1:1,000, 21645-1-AP, Proteintech), RhoA (1:500, 10749-1-AP, Proteintech), Beclin1 (1:1,000, 11306-1-AP, Proteintech), microtubule-associated protein 1 light chain (LC3) (1:1,000, ab48394, Abcam) and β-actin (1:1,000, 20536-1-AP, Proteintech) overnight at 4°C. Then the membranes were incubated with horseradish peroxidase-conjugated secondary antibodies (1:5,000, GB23303, Servicebio) for 1h at room temperature. Finally, the expressions of target protein were analyzed with ECL kit and Exposure System (Bio-Rad Laboratories) and normalized to the β-actin for semiquantification.

### Immunohistochemistry

After fixation and embedding in paraffin, 5-μm-thick sections of the corpus cavernosum were incubated with GLP-1R antibody (1:200, A13990, Abclonal) followed by a biotinylated secondary antibody to decide the expression and location of the target protein. The persons performing immunohistochemistry had been blinded with regard to group allocation. After that, a semiquantitative analysis was conducted by measuring the mean optical density with Image-Pro plus software 6.0.

### Fluorescence Staining

Similar to immunohistochemistry, the location and expression levels of LC3 in the corpus cavernosum were measured by incubating tissue slides with anti-LC3 (1:200, ab48394, Abcam) followed by corresponding FITC-conjugated secondary antibody. In addition, DAPI (4’,6-diamidino-2-phenylindole, Sigma-Aldrich) was used to stain the cell nuclei. Also, the persons performing fluorescence staining had been blinded with regard to group allocation.

ROS in the corpus cavernosum was detected using a fluorescent probe (Dihydroethidium, DHE; Beyotime Biotechnology). The tissue slides were incubated with DHE at room temperature for half an hour. DAPI was used to stain the cell nuclei. Then they were observed under a fluorescence microscope image. The results were analyzed by averaging fluorescence intensity of photographs using Image-Pro Plus software 6.0.

*In vitro* study, CCSMCs were cultured in 6-well plates. After reaching 70%–80% confluent, they were fixed with 4% formaldehyde and treated with DHE or LC3 immunofluorescence staining in similar protocols of the corpus cavernosum.

### Examination of Oxidative Activity

The levels of malondialdehyde (MDA) reflecting oxidative activities and superoxide dismutase (SOD) reflecting antioxidant activities in the corpus cavernosum were examined by assay kits (S0131 for MDA and S0101 for SOD, Beyotime Biotechnology) according to corresponding protocols.

### Statistical Analysis

The photograph data were analyzed based on the fluorescence intensity or grayscale by Image-Pro Plus 6.0 software. One-way ANOVA analysis and Tukey’s test for *post hoc* comparisons were conducted to compare differences among groups by Statistical Package for Social Science (SPSS 23.0). P < 0.05 was regarded as statistical significance. Final results were shown as figures by GraphPad Prism 8.0.

## Results

### Metabolic Indices in Rats

**Figure 1:**
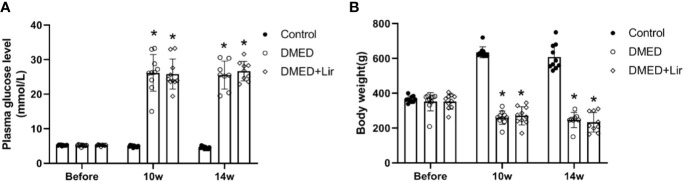
Metabolic parameters in rats. **(A)** Time course of fasting blood glucose (mmol/L) measurements. **(B)** Time course of body weight (g) measurements. Before means the initial body weight and plasma glucose level measured after 7-day adaptive feeding. Liraglutide was treated from the 10th to the 14th week. Data are expressed as means ± SD. *P < 0.05 vs. the control group. DMED, diabetes mellitus-induced erectile dysfunction; Lir, liraglutide.

The body weight and fasting blood glucose of rats were recorded during the animal experiment. There was no significant difference in initial levels of fasting blood glucose and body weight among all of the groups (p > 0.05, **Figures 1A, B**). At the end of 10th and 14th week after diabetes induction, rats in the DMED and DMED + Lir groups showed higher blood glucose levels and lower body weights compared with those in the control group significantly (p < 0.05), which suggested a successful establishment of the rat model of type 1 DM. However, there was no significant difference in the final blood glucose levels and body weights between the DMED and DMED + Lir groups after the 4-week injection of liraglutide.

### Assessment of Erectile Function in Rats

**Figure 2:**
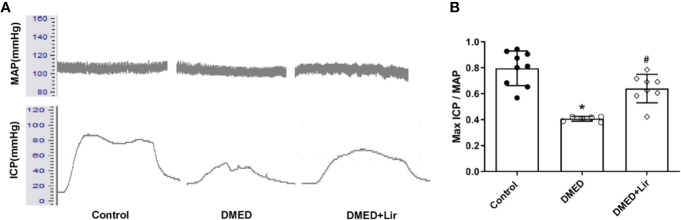
Assessment of erectile function in rats. **(A)** Representative traces of ICP and MAP (stimulation parameters: 15-Hz frequency; 1.2-ms width; 5.0 V). **(B)** Max ICP/MAP among the groups. Data are expressed as means ± SD. *P < 0.05 vs. the control group; #P < 0.05 vs. the DMED group. DMED, diabetes mellitus-induced erectile dysfunction; Lir, liraglutide; ICP, intracavernous pressure; MAP, mean atrial blood pressure.

The erectile function was quantified based on max ICP and MAP. It indicated that the value of max ICP/MAP was much lower in the DMED group compared with that in the control group (**Figures 2A, B**; p < 0.05). And the administration of liraglutide made the value of max ICP/MAP of DMED + Lir group significantly higher than that in the DMED group (p < 0.05), which suggested a therapeutic role of liraglutide in ED.

### Effects of Liraglutide on Reducing Oxidative Stress *In Vivo* and *In Vitro*

**Figure 3:**
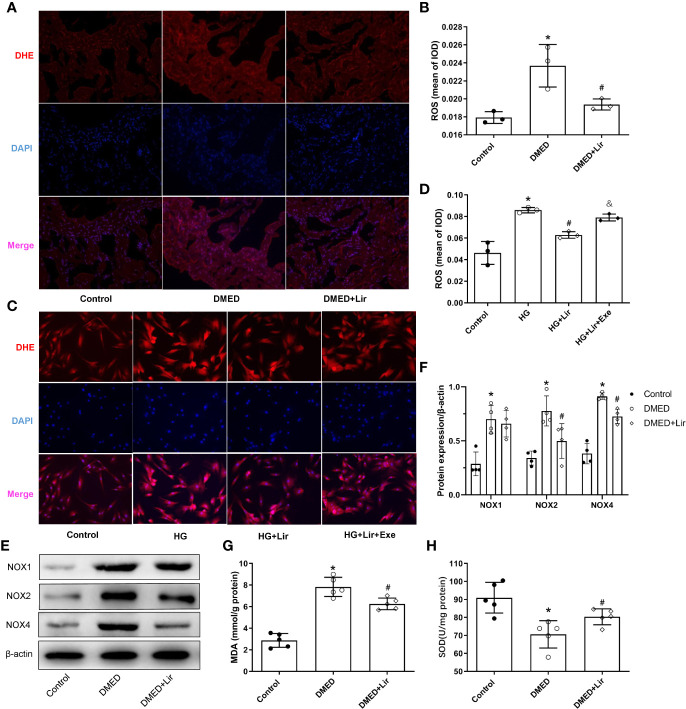
Effects of liraglutide on reducing oxidative stress *in vivo* and *in vitro*. Representative immunofluorescence (×200) **(A)** and quantification **(B)** of ROS among the groups in the corpus cavernosum. Representative immunofluorescence (×200) **(C)** and quantification **(D)** of ROS among the groups in CCSMCs. Representative immunoblot **(E)** and quantification **(F)** of NOX1, NOX2 and NOX4 among the groups in the corpus cavernosum. Levels of MDA **(G)** and SOD **(H)** among the groups in the corpus cavernosum. The quantitative data of β-actin in 3F: 1, 1.16, and 1.25, respectively. Data are expressed as means ± SD. *P < 0.05 vs. the control group; #P < 0.05 vs. the DMED group or the HG group; & P < 0.05 vs. the HG + Lir group. CCSMCs, corpus cavernosum smooth muscle cells; DMED, diabetes mellitus-induced erectile dysfunction; Lir, liraglutide; HG, high glucose; Exe, Exendin (9-39); ROS, reactive oxygen species; DHE, Dihydroethidium; DAPI, 4’,6-diamidino-2-phenylindole; NOX, nicotinamide adenine dinucleotide phosphate (NADPH) oxidase; MDA, malondialdehyde; SOD, superoxide dismutase; IOD, Integrated option density.

Primary cultured CCSMCs were identified by double immunofluorescence labeling of α-SMA (green) and desmin (red) (**Supplementary Figure 1**). The fact that almost all the cells showed positive results indicated that the degree of cellular purity was suitable for further studies *in vitro*. ROS is an important pathological factor in the occurrence of DMED, and the main function of NOXs is to transfer electrons across the plasma membrane to molecular oxygen and result in the generation of ROS (Bedard and Krause, 2007). DHE staining reflecting ROS was conducted in the corpus cavernosum and CCSMCs. The results showed that ROS expression was more obvious in the DMED group (p < 0.05, **Figures 3A, B**), and liraglutide treatment attenuated ROS production significantly (p < 0.05). Similarly, ROS expression in HG + Lir group was notably downregulated compared with that in the HG group *in vitro* (p < 0.05, **Figures 3C, D**). Furthermore, we examined the protein expression levels of NOXs in the corpus cavernosum. The expression levels of NOX2 and NOX4 were higher in the DMED group than those in the control group (p < 0.05, **Figures 3E, F**), while DMED + Lir group showed lower levels of NOX2 and NOX4 compared with those in the DMED group (p < 0.05). Then MDA and SOD measurements indicated a similar trend. Increased MDA level and decreased SOD level existed in the DMED group (p < 0.05, **Figures 3G, H**). These changes were partly but remarkably reduced by liraglutide (p < 0.05).

### Effects of Liraglutide on Regulating the RhoA/ROCK Pathway *In Vivo* and *In Vitro*

**Figure 4:**
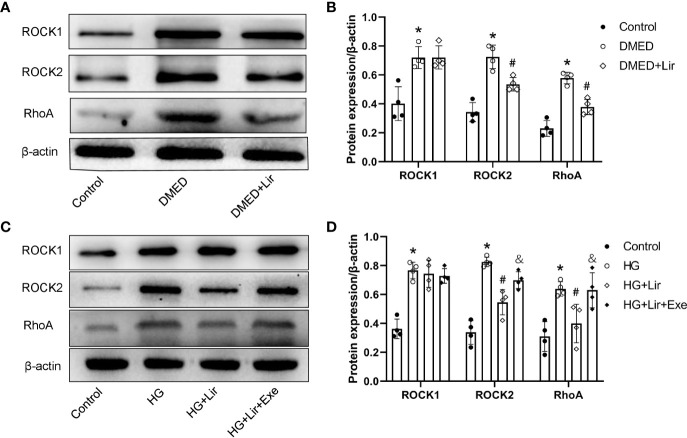
Effects of liraglutide on regulating RhoA/ROCK pathway *in vivo* and *in vitro*. Representative immunoblot **(A)** and quantification **(B)** of ROCK1, ROCK2 and RhoA among the groups in the corpus cavernosum. Representative immunoblot **(C)** and quantification **(D)** of ROCK1, ROCK2 and RhoA among the groups in CCSMCs. The quantitative data of β-actin: 1, 1.15, and 0.91, respectively in 4B and 1, 1.04, 1.19, and 1.28, respectively in 4D. Data are expressed as means ± SD. *P < 0.05 vs. the control group; ^#^P < 0.05 vs. the DMED group or the HG group; ^&^P < 0.05 vs. the HG + Lir group. CCSMCs, corpus cavernosum smooth muscle cells; DMED, diabetes mellitus-induced erectile dysfunction; Lir, liraglutide; HG, high glucose; Exe, Exendin (9-39); RhoA, Ras homolog gene family; ROCK, Rho-associated protein kinase.

Downregulation of the RhoA/ROCK signaling pathway is involved in penile erection by regulating the contractile state of CCSMCs. Western blotting analysis *in vivo* demonstrated that expression levels of RhoA, ROCK1 and ROCK2 were markedly increased in the DMED group than in the control group (p < 0.05, **Figures 4A, B**). After liraglutide administration, evident decreases in RhoA and ROCK2 were shown in the DMED + Lir group (p < 0.05). This trend was also seen in CCSMCs after administration of HG and liraglutide (p < 0.05, **Figures 4C, D**).

### Effects of Liraglutide on Regulating Autophagy *In Vivo* and *In Vitro*

**Figure 5:**
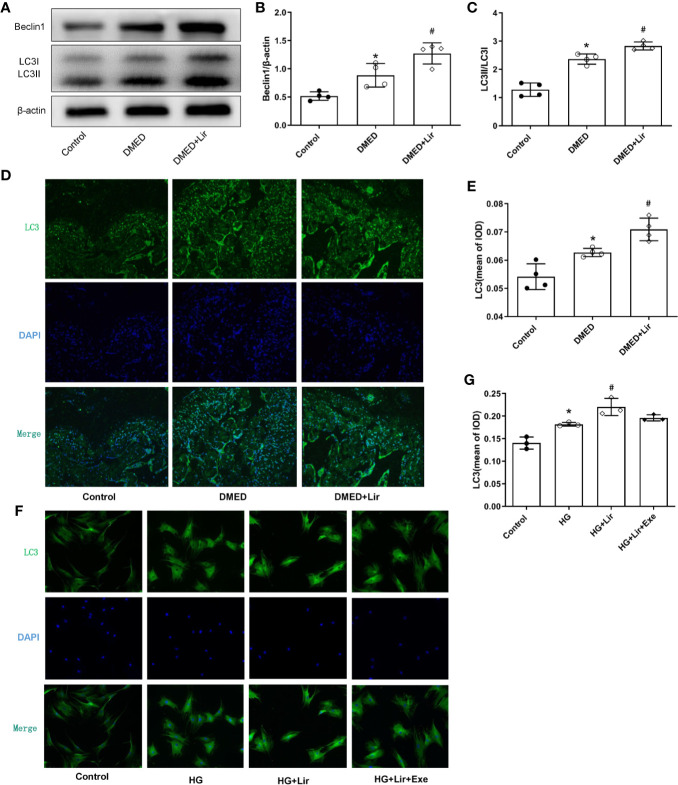
Effects of liraglutide on regulating autophagy *in vivo* and *in vitro*. Representative immunoblot **(A)** and quantification **(B, C)** of Beclin1 and LC3II/LC3I among the groups in the corpus cavernosum. Representative immunofluorescence (×200) **(D)** and quantification **(E)** of LC3 among the groups in the corpus cavernosum. Representative immunofluorescence (×200) **(F)** and quantification **(G)** of LC3 among the groups in CCSMCs. The quantitative data of β-actin in 5B: 1, 1.18 and 1.32, respectively. Data are expressed as means ± SD. *P < 0.05 vs. the control group; #P < 0.05 vs. the DMED group or the HG group. CCSMCs, corpus cavernosum smooth muscle cells; DMED, diabetes mellitus-induced erectile dysfunction; Lir, liraglutide; HG, high glucose; Exe, Exendin (9-39); LC3, microtubule-associated protein 1 light chain; DAPI, 4’,6-diamidino-2-phenylindole; IOD, Integrated option density.

To investigate the effects of autophagy underpinning in erectile function, the protein expression levels and distributions of autophagy associated proteins were examined. In western blotting analysis, elevated expressions of Beclin 1 and autophagy marker LC3 were observed in the DMED + Lir group compared with the DMED group (p < 0.05, **Figures 5A–C**). Furthermore, immunofluorescence labeling of LC3 was conducted to explore autophagy in the corpus cavernosum and CCSMCs. It showed that green LC3 expression in the corpus cavernosum was significantly higher in the DMED group than that in the control group (p < 0.05, **Figures 5D, E**), and the trend was even more pronounced in the DMED+ Lir group (p < 0.05). These results were consistent with studies *in vitro* (**Figures 5F, G**).

### Effects of Liraglutide on the Expression of GLP-1R *In Vivo* and *In Vitro*

**Figure 6:**
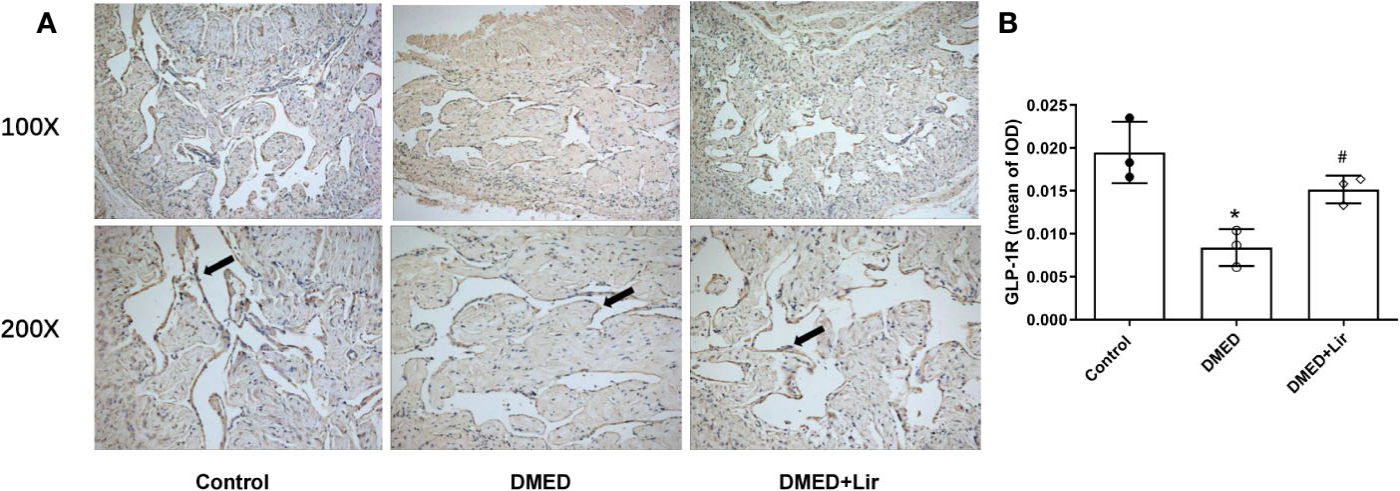
Effects of liraglutide on expression of GLP-1R in the corpus cavernosum. Representative immunohistochemistry **(A)** and quantification **(B)** of GLP-1R among the groups. Data are expressed as means ± SD. *P < 0.05 vs. the control group; #P < 0.05 vs. the DMED group. DMED, diabetes mellitus-induced erectile dysfunction; Lir, liraglutide; GLP-1R, glucagon-like peptide-1 receptor; IOD, Integrated option density.

GLP-1 is generally believed to exert its actions through GLP-1R, which was thought expressed in endothelium and vascular smooth muscle cells. Thus, immunohistochemistry of corpus cavernosum was conducted to compare the GLP-1R expression among the three groups. It suggested that GLP-1R expression was decreased significantly in the corpus cavernosum of DMED group compared with the control group, while liraglutide treatment improved this condition dramatically (p < 0.05, **Figures 6A, B**). Furthermore, administration of the GLP-1R antagonist exendin (9–39) *in vitro* ameliorated the effects of liraglutide on CCSMCs under HG condition, including increased ROS (p < 0.05) (**Figures 3C, D**) and activated RhoA/ROCK pathway (p < 0.05) (**Figures 4C, D**).

## Discussion

DM and its complications are important health problems and are concerned globally. Erectile function impairment is more common in diabetic men. However, effective medications to prevent this phenomenon are limited. GLP-1 is a kind of gut hormone secreted in a nutrient-dependent manner and involved in the regulation of the secretion of insulin and glucagon in the pancreas (Inoue et al., 2015). Nowadays GLP-1 analogs have been severed as hypoglycemic drugs for type 2 diabetes. Besides, more and more studies focus on the extra-pancreatic functions of GLP-1. Cumulative evidence has expounded the role of GLP-1 in the cardiovascular system. As part of the vasculature, it is significant to expound the function of GLP-1 in the corpus cavernosum. A retrospective study showed that GLP-1 analog liraglutide supplement resulted in significant improvements of the IIEF (International Index of Erectile Function) -15 scores compared with nonliraglutide users in patients with poor glycemic response (Giagulli et al., 2015). However, the underlying mechanism was not fully illuminated since liraglutide ameliorated abnormal metabolic indices in diabetics, including blood glucose and body weights, which are important risk factors for ED. Whether liraglutide plays a direct role in ED is unknown. In this study, we used the rats with STZ-induced diabetes. The results demonstrated that the liraglutide treatment did not affect body weights and plasma glucose levels significantly, but it still improved erectile function with significantly higher ICPmax/MAP value compared with that in DMED rats. Based on this, we preliminarily confirmed that liraglutide ameliorated ED through a glucose or weight independent manner. Then we explored the underlying mechanisms behind the protective effect further *in vivo* and *in vitro*.

Oxidant injury induced by diabetes is the key mechanism in DMED (Neves, 2013). In diabetes, long-term hyperglycemia stimulation induces AGEs formation, which triggers ROS accumulation excessively. The latter could trigger a series of biological processes influencing vasoconstriction, modifying the extracellular matrix and destructing cellular components, ultimately leading to target organ dysfunction (Sayre et al., 2001). During the occurrence of DMED, oxidative stress could decrease intracavernous blood flow, interfere endothelial-dependent vasodilation (Neves, 2013), impair the corpus cavernosum muscle relaxation and result in erectile tissue remodeling and degeneration (Azadzoi et al., 2005). Song et al. (Song et al., 2019) reported that ROS played an important role in the pathophysiology of DMED. Azadzoi et al. (2005) demonstrated that oxidative products were accumulated in the erectile tissue during the process of ED and antioxidant therapy could serve as a significant tool for preventing smooth muscle dysfunction in ED. In this study, we examined the changes in oxidative stress in the corpus cavernosum and smooth muscle cells. It showed that NOXs, the major sources of ROS generated in the vessel wall, were significantly upregulated in the DMED group. Consistent with these, the lower SOD activity and higher MDA activity existed in diabetic rats. Studies showed that liraglutide exerted a protective role in diabetic nephropathy and cardiomyopathy against oxidative stress (Hendarto et al., 2012; Inoue et al., 2015). In this experiment, ROS production and NOXs levels were significantly decreased in the liraglutide treatment group, suggesting a therapeutic effect in ED.

RhoA/ROCK signaling pathway regulates multiple biological functions throughout the body (Breitenlechner et al., 2003; Priviero et al., 2010). Of them, the importance of the RhoA/ROCK pathway in maintaining a flaccid penile state is concerned (Sopko et al., 2014). In diabetic ED, excessive accumulation of ROS activated the RhoA/ROCK signaling pathway furthermore (Lu et al., 2013), which is the reason we looked into the RhoA/ROCK pathway after the awareness of the protect effect of liraglutide on the corpus cavernosal tissue from diabetes-relative oxidative stress by the ROS test. During the contracting process of cavernosum smooth muscle, a small monomeric GTPase, RhoA, activates ROCK, which makes MLCP phosphorylated and promotes penile contraction ultimately (Sopko et al., 2014). Morelli et al. (2009) showed that human cavernosum smooth muscle cells exposed to high-glucose condition led to the activation of RhoA/ROCK activity. And cavernosal tissues in diabetic mice had elevated levels of RhoA and ROCK2 (Toque et al., 2013). Furthermore, ROCK signaling inhibitor significantly reduced the contractile response and elevated the endothelium-dependent relaxation in diabetic rat aorta rings (Cicek et al., 2013). In the present study, higher expression levels of RhoA and ROCK2 were observed in the DMED rats. After liraglutide administration, oxidative stress was ameliorated accompanied by the downregulation of the RhoA/ROCK pathway. These preliminarily indicated that liraglutide ameliorated ED *via* the RhoA/ROCK2 pathway.

Autophagy is a conserved cellular metabolic process that maintains homeostasis in organisms by the degradation of damaged or dysfunctional proteins and organelles *via* lysosomes under unfavorable conditions such as oxidative stress (Klionsky, 2007). Wang et al. (2015) showed that castration impaired erectile issue structure and function by inhibiting autophagy. Lin et al. (2018) found a higher autophagy level in rats with STZ-induced diabetes. Previous studies found that liraglutide relieved cognitive decline and myocardial damage by promoting autophagy *via* the AMP-activated protein kinase/mammalian target of rapamycin pathway (Zhang et al., 2017; Kong et al., 2018b). Based on these, we examined the expression of autophagy markers in the corpus cavernosal tissue and CCSMCs to expound the role of autophagy in the protective effect of liraglutide,. The results revealed that enhanced levels of autophagy associated proteins Beclin 1 and the ratio of LC3II and LC3I were noted in the DMED group. And liraglutide treatment further promoted autophagy by exhibiting higher expression of autophagy markers and the percentage of autophagy cells labeled by LC3/DAPI. That’s an interesting result. As a matter of fact, moderate autophagy functions to maintain normal cellular function. During the process of diabetic ED, excessive ROS accumulation would induce compensatory autophagy to protect against oxidative stress by eliminating damaged intracellular materials (Scherz-Shouval et al., 2007). However, the extent was far from enough to counteract harmful conditions and restore the homeostasis of the body in DMED (Ding et al., 2020). And the administration of liraglutide ameliorated metabolic disturbance by regulating autophagy to a large extent.

GLP-1 is thought to exert its functions through GLP-1R (Cai et al., 2017). To explore the receptor-dependent mechanism of liraglutide on erectile function, we examined the expression profiles of GLP-1R by immunohistochemistry in the corpus cavernosum of all groups. It showed that GLP-1R expression was decreased in diabetic issues, while administration of liraglutide could partially restore this condition significantly. Furthermore, CCSMCs were isolated and cultured in HG-containing medium with or without liraglutide and GLP-1R inhibitor. In line with results *in vivo*, liraglutide inhibited ROS production, accompanied by regulation of RhoA/ROCK pathway and autophagy in high-glucose condition. While GLP-1R inhibitor abolished the beneficial functions of liraglutide treatment effectively.

Based on these, we expounded the protective role of liraglutide in ED. Oxidative stress caused by ROS is a predominant factor in the pathogenesis of DMED, which could activate the RhoA/ROCK pathway to inhibit vasodilatation. Furthermore, compensatory autophagy enhances to counteract the damage caused by oxidative stress, though the autophagy level is not enough to maintain homeostasis. And liraglutide could improve erectile function by alleviating oxidative stress as well as regulation of the RhoA/ROCK pathway and autophagy.

There were some limitations in this study. First, to confirm the direct benefit of liraglutide on erectile function, we conducted experiments in STZ-induced type 1 diabetes, which impeded generalization to the general diabetic population. In addition, although we revealed the changes of relevant proteins in erectile issues after liraglutide treatment, the specific mechanisms behind the causality between oxidative stress, autophagy and liraglutide were not clear enough. And the interactions between GLP-1R and these effects need also exploring. In future studies, we will explore the detailed processes in which GLP-1 and its receptor regulate erectile function in diabetes.

The results show that liraglutide exerts protective effects on ED associated with the regulation of smooth muscle dysfunction, oxidative stress and autophagy, independently of a glucose lowering effect. The results enhance our understanding of pathological changes of DMED and provide new insights into the extrapancreatic actions of liraglutide. It provides preclinical evidence for a potential treatment for DMED.

## Data Availability Statement

The raw data supporting the conclusions of this article will be made available by the authors, without undue reservation.

## Ethics Statement

The animal study was reviewed and approved by Experimental Animal Administration Committee of Wuhan Servicebio Biotechnology.

## Author Contributions

Conceptualization: PY and XL. Formal analysis: PY and DM. Writing—original draft: PY and XL. Funding acquisition: XL and DM. Data curation: DM, XG, and JW. Investigation: RL and ZL. Resources: TW. Writing—review and editing: PY, DM, TW, SW, JL, and XL. Supervision: JL and XL.

## Funding

This work was supported by the National Natural Science Foundation of China (Grant Number: 81702518), Huazhong University of Science and Technology (Grant Number: 2019kfyXKJC06), and National Natural Science Foundation of China (Grant Number: 81500636).

## Conflict of Interest

The authors declare that the research was conducted in the absence of any commercial or financial relationships that could be construed as a potential conflict of interest.
