# Supplementary material for: Liraglutide Ameliorates Erectile Dysfunction via Regulating Oxidative Stress, the RhoA/ROCK Pathway and Autophagy in Diabetes Mellitus
Source: Front Pharmacol. 2020 Aug 12;11:1257. doi: 10.3389/fphar.2020.01257 (PMC7435068; doi:10.3389/fphar.2020.01257)
Supplement: Supplementary file 1 [file Image_1.pdf]

Supplementary Material

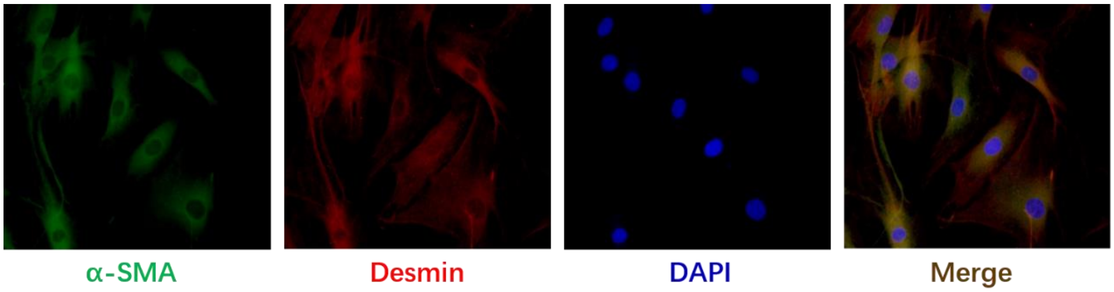

Supplementary Figure 1. Identification of CCSMCs by double immunofluorescence labeling (×200). CCSMCs = corpus cavernosum smooth muscle cells; α-SMA = α-smooth muscle actin; DAPI = 4’,6-diamidino-2-phenylindole.
